# Supplementary figures and images for: Comparison of GWAS models to identify non-additive genetic control of flowering time in sunflower hybrids
Source: Theor Appl Genet. 2017 Nov 2;131(2):319–32. doi: 10.1007/s00122-017-3003-4 (PMC5787229; doi:10.1007/s00122-017-3003-4)

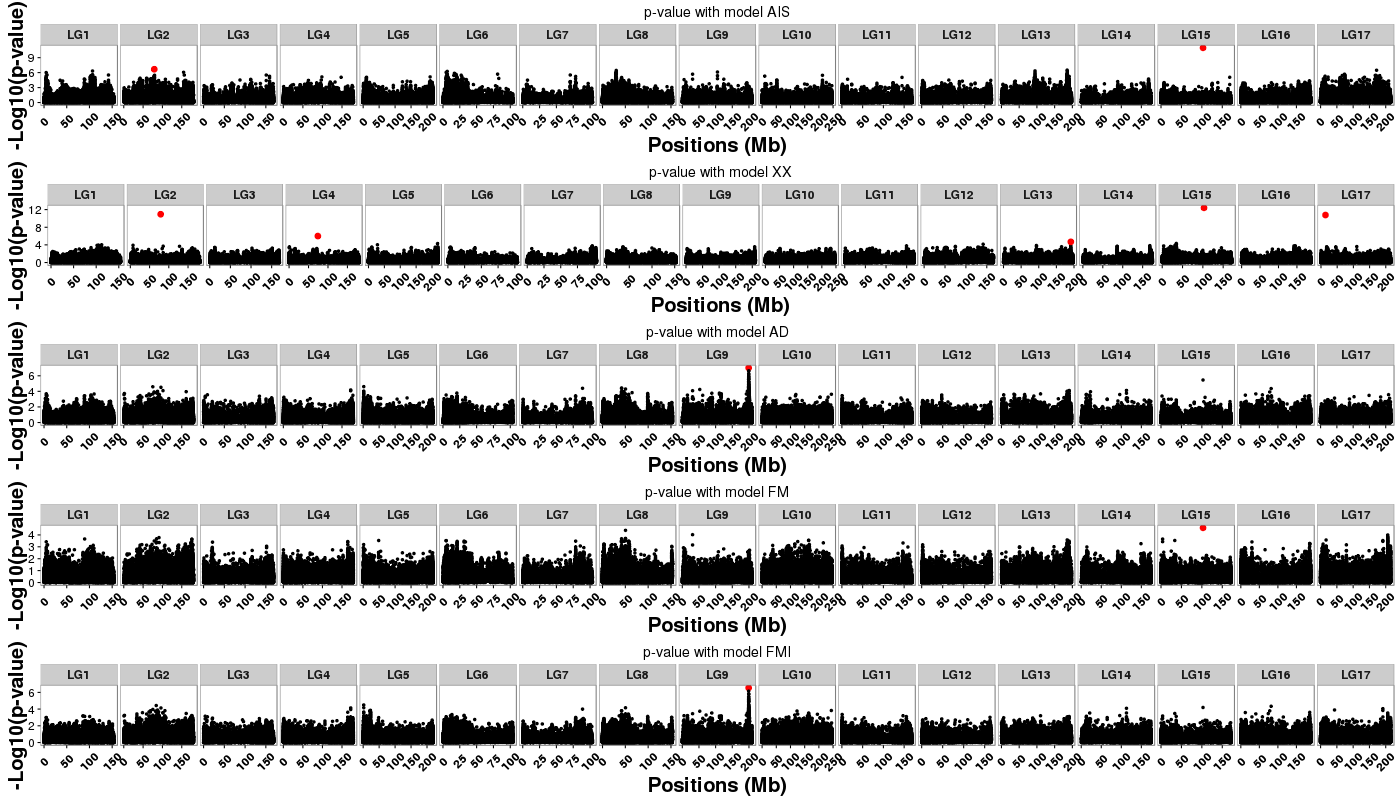

Supplement: Supplementary file 3 — Supplementary material 3 (png 181 KB) [file 122_2017_3003_MOESM3_ESM.png]

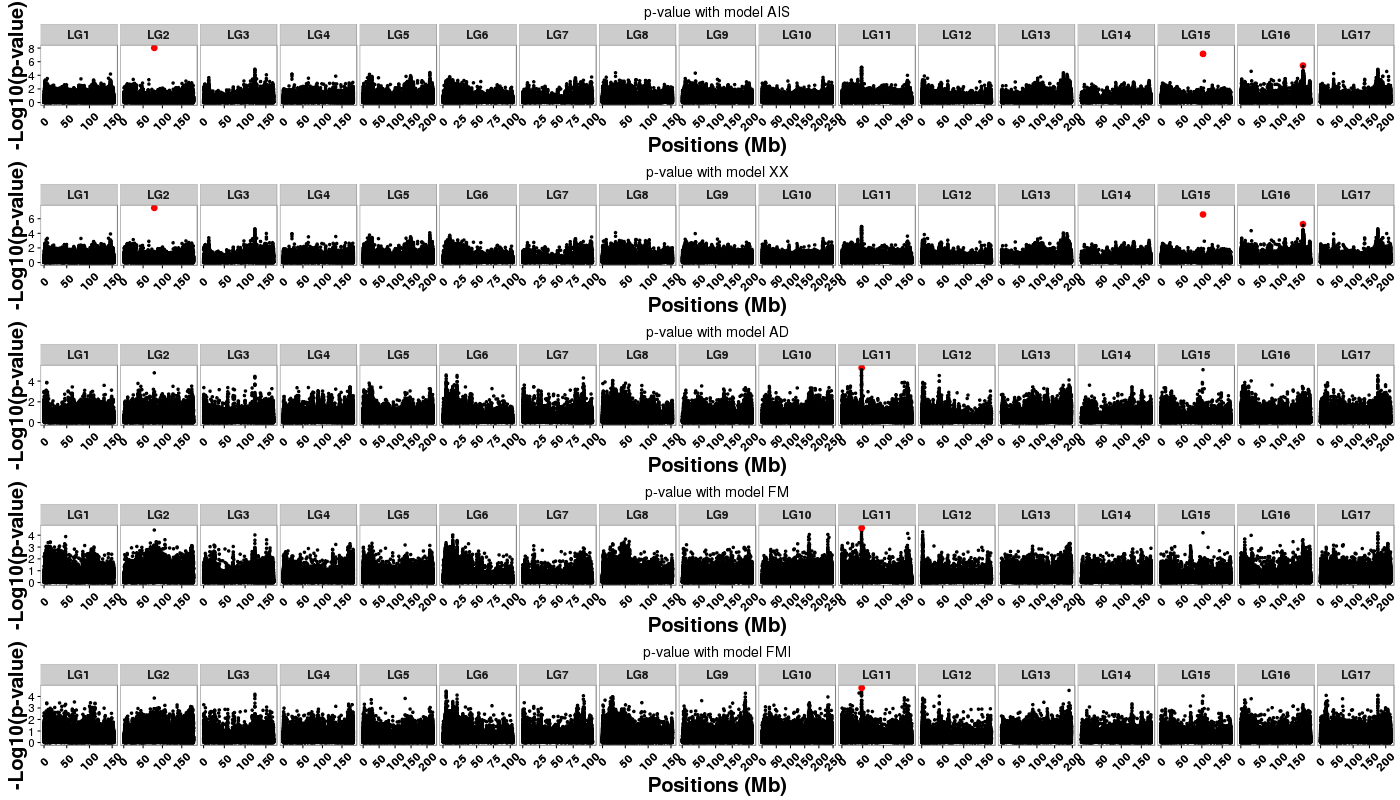

Supplement: Supplementary file 4 — Supplementary material 4 (png 201 KB) [file 122_2017_3003_MOESM4_ESM.png]

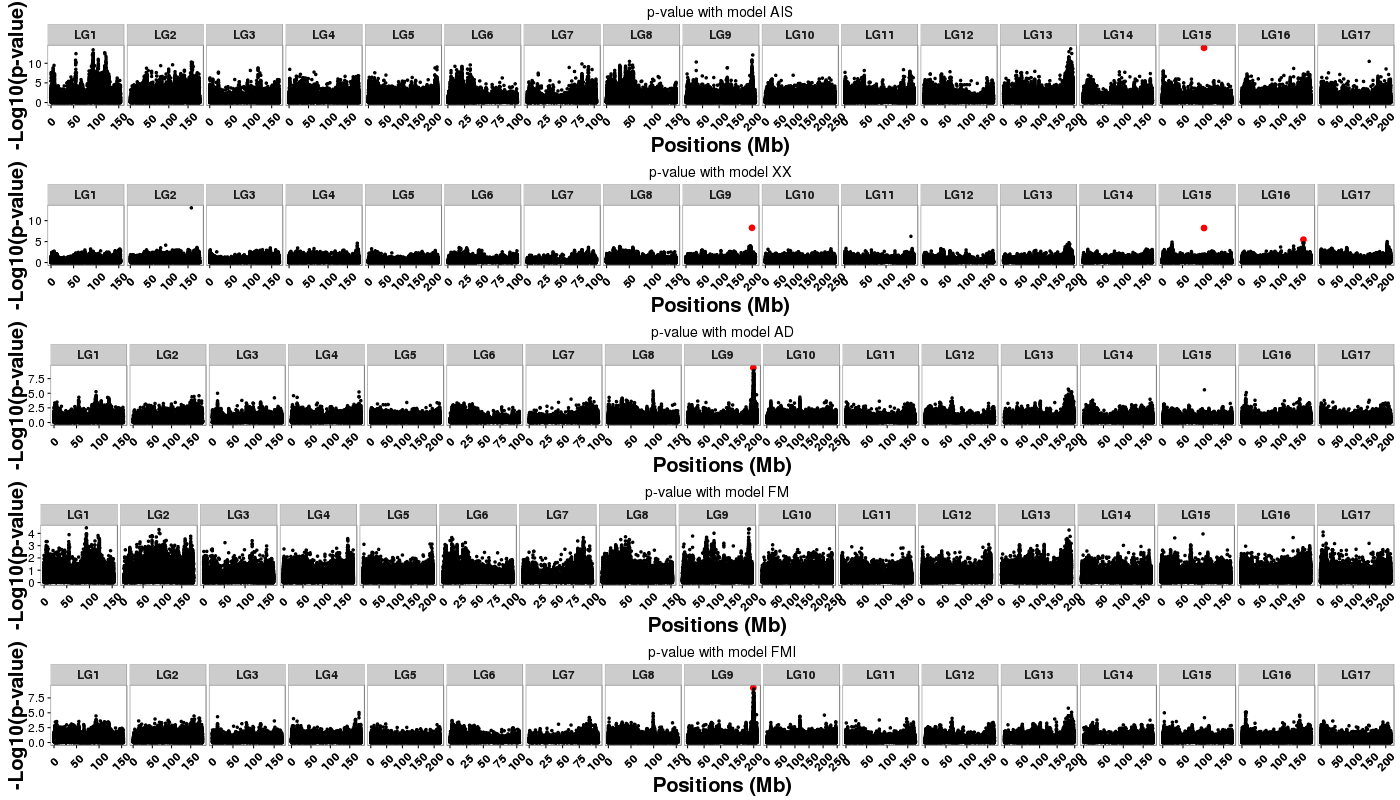

Supplement: Supplementary file 5 — Supplementary material 5 (png 180 KB) [file 122_2017_3003_MOESM5_ESM.png]

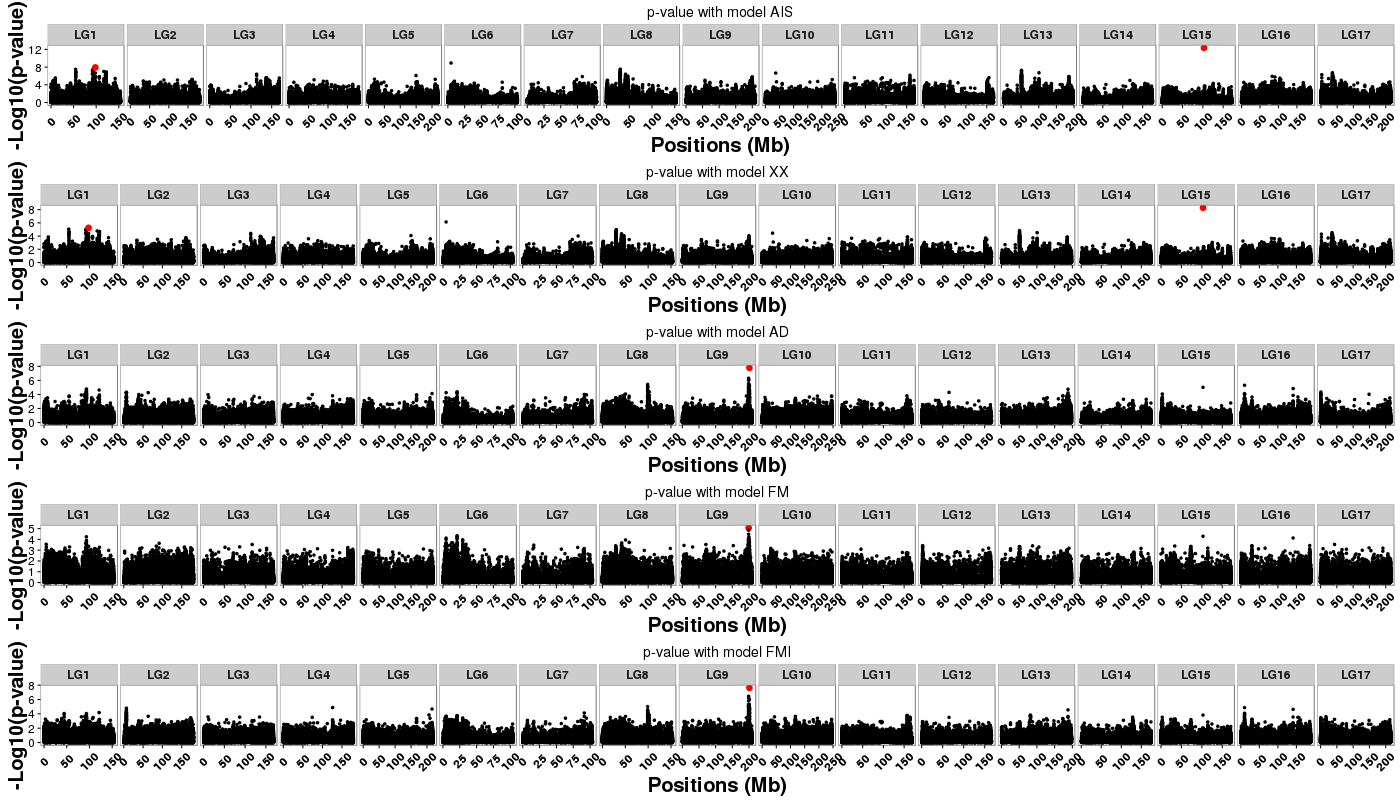

Supplement: Supplementary file 6 — Supplementary material 6 (png 186 KB) [file 122_2017_3003_MOESM6_ESM.png]

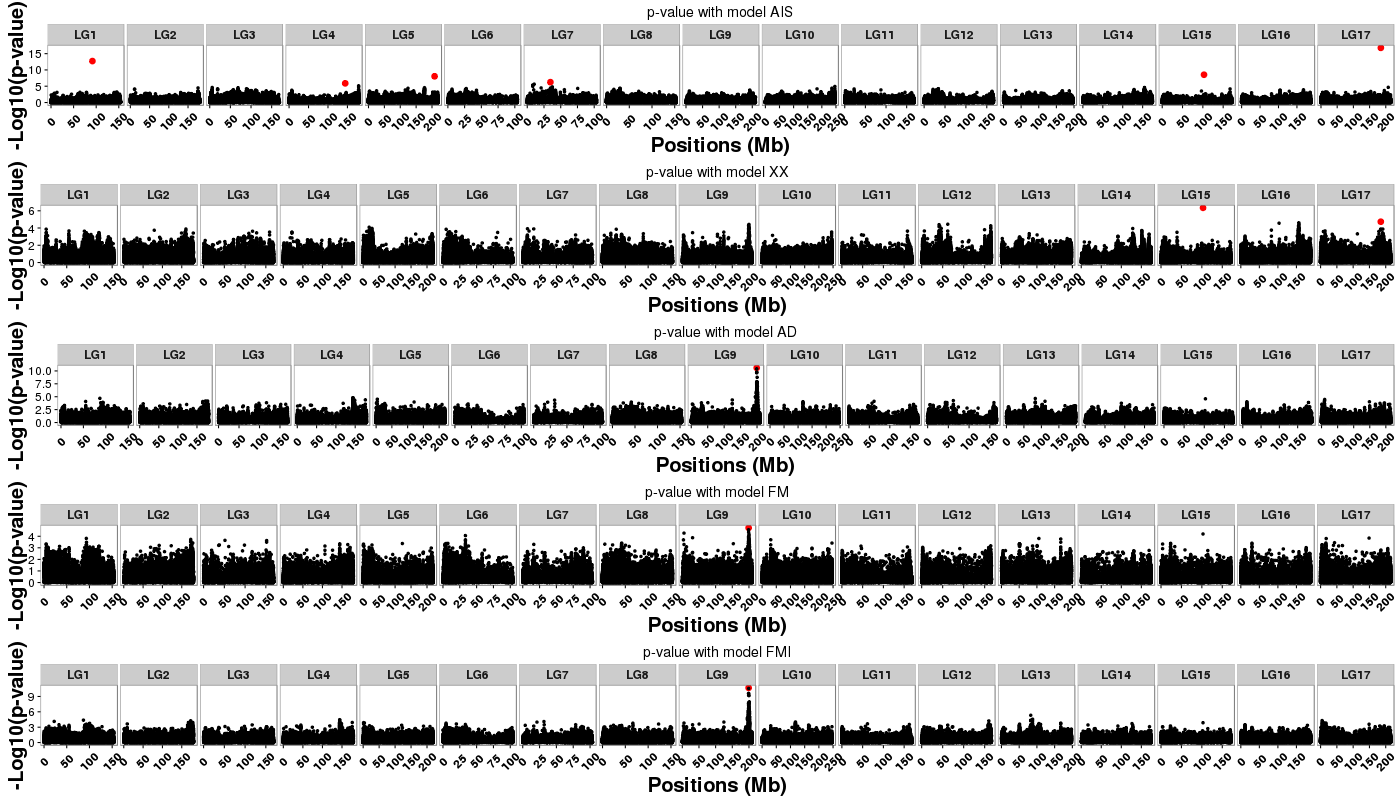

Supplement: Supplementary file 7 — Supplementary material 7 (png 175 KB) [file 122_2017_3003_MOESM7_ESM.png]
