# Supplementary material for: Comparison of GWAS models to identify non-additive genetic control of flowering time in sunflower hybrids
Source: Theor Appl Genet. 2017 Nov 2;131(2):319–32. doi: 10.1007/s00122-017-3003-4 (PMC5787229; doi:10.1007/s00122-017-3003-4)
Supplement: Supplementary file 9 — Supplementary material 9 (pdf 150 KB) [file 122_2017_3003_MOESM9_ESM.pdf]

Noname manuscript No.
(will be inserted by the editor)

Comparison of GWAS models to identify non-additive genetic control of flowering time in sunflower hybrids

Fanny Bonnafous · Ghislain Fievet · Nicolas Blanchet · Marie-Claude Boniface · Sébastien Carrère · Jérôme Gouzy · Ludovic Legrand · Gwenola Marage · Emmanuelle Bret-Mestries · Stéphane Munos · Nicolas Pouilly · Patrick Vincourt · Nicolas Langlade · Brigitte Mangin

Received: date / Accepted: date

F. Bonnafous
LIPM, Universit de Toulouse, INRA, CNRS, Castanet-Tolosan, France
Tel.: +33 5 61 28 57 27
E-mail: fanny.bonnafous@inra.fr

G. Fievet · N. Blanchet · M-C. Boniface · S. Carrère · J. Gouzy · L. Legrand · G. Marage · S. Munos · N. Pouilly · P. Vincourt · N. Langlade · B. Mangin
LIPM, Université de Toulouse, INRA, CNRS, Castanet-Tolosan, France

E. Bret-Mestries
TERRES INOVIA, AGIR, Castanet-Tolosan, France

Fig. S1: Heatmap of phenotypic values per environment (13EX01 to 13EX06). For each heatmap (one environment), the flowering time values are illustrated by hybrid (one square) according to their parents (female lines on the left and male lines at the bottom). Parents are ranked by the mean of their descendants. The more the square are green, the earlier the hybrid has bloomed, and the more the square are red, more the hybrid has bloomed late.

Fig. S2: Flowering time in the sunflower hybrid population across environments. Pearsons correlation values are in red on the top right, trait distribution for each environment is presented in gray (middle), and point clouds with the regression slope (red line) with confidence interval (green shading) are presented on the bottom left. Correlations between two environments are based on 370 hybrids on average (minimum 297, maximum 425).

Fig. S3: Manhattan plot for all models (A_{AIS}, A_{XX}, AD, FM and FMI) for the environment 13EX01. For each model, the p-values off all SNPs of the analysis are represented according to the position of the SNPs on the genome (in Mb). P-values are those of the step of the algorithm where all SNPs that are part of a QTL (red points) have been detected with the model of interest.

Fig. S4: Manhattan plot for all models (A_{AIS}, A_{XX}, AD, FM and FMI) for the environment 13EX02. For each model, the p-values off all SNPs of the analysis are represented according to the position of the SNPs on the genome (in Mb). P-values are those of the step of the algorithm where all SNPs that are part of a QTL (red points) have been detected with the model of interest.

Fig. S5: Manhattan plot for all models (A_{AIS}, A_{XX}, AD, FM and FMI) for the environment 13EX03. For each model, the p-values off all SNPs of the analysis are represented according to the position of the SNPs on the genome (in Mb). P-values are those of the step of the algorithm where all SNPs that are part of a QTL (red points) have been detected with the model of interest.

Fig. S6: Manhattan plot for all models (A_{AIS}, A_{XX}, AD, FM and FMI) for the environment 13EX04. For each model, the p-values off all SNPs of the analysis are represented according to the position of the SNPs on the genome (in Mb). P-values are those of the step of the algorithm where all SNPs that are part of a QTL (red points) have been detected with the model of interest.

Fig. S7: Manhattan plot for all models (A_{AIS}, A_{XX}, AD, FM and FMI) for the environment 13EX06. For each model, the p-values off all SNPs of the analysis are represented according to the position of the SNPs on the genome (in Mb). P-values are those of the step of the algorithm where all SNPs that are part of a QTL (red points) have been detected with the model of interest.

Fig. S8: Manhattan plot of p-values of SNPs in QTL FT09.199. Combination of the association model and environment when significant association were found are illustrated. P-values are calculated in the corresponding model with the EMMAX model approximation. Red lines indicate the positions of associated SNPs. Gene involved in flowering process are positioned and indicated in red.

Table S1: Parts of variance for the models A ($A_{XX'}$), AD, FM and FMI for all the environments (13EX01 to 13EX06). σ_a^2, σ_d^2, σ_f^2, σ_m^2, σ_{fm}^2 and σ_e^2 represent the additive, the dominant, the female, the male, the interaction between female and male, and the residual part of variances, respectively. The model used to compute theses parts of variances is the one without the effects of SNPs, using only kinships. The values of the variance components are shown in parentheses.

		13EX01	13EX02	13EX03	13EX04	13EX06
A	σ_a^2	0.19 (331.47)	0.19 (257.83)	0.21 (540.81))	0.19 (538.11)	0.19 (399.45)
	σ_e^2	0.81 (1405.78)	0.81 (1115.82)	0.79 (2080.87)	0.81 (2258.57)	0.81 (1663.83)
AD	σ_a^2	0.17 (333.85)	0.17 (263.37)	0.20 (509.98)	0.19 (514.87)	0.20 (384.92)
	σ_d^2	0.15 (279.36)	0.16 (239.70)	0.16 (40.41)	0.01 (13.38)	0.01 (9.86)
	σ_e^2	0.68 (1301.48)	0.67 (1031.70)	0.64 (1947.71)	0.80 (2115.86)	0.79 (1559.95)
FM	σ_f^2	0.30 (585.79)	0.28 (456.95)	0.37 (1321.31)	0.32 (1178.76)	0.33(866.58)
	σ_m^2	0.40 (777.16)	0.41 (660.31)	0.46 (1644.47)	0.44 (1617.84)	0.43 (1118.92)
	σ_e^2	0.30 (574.80)	0.31 (505.34)	0.17(635.86)	0.24 (846.81)	0.24 (651.70)
FMI	σ_f^2	0.30 (576.35)	0.29 (472.04)	0.36 (1332.79)	0.32 (1178.76)	0.33 (870.75)
	σ_m^2	0.42 (817.42)	0.42 (693.70)	0.47 (1719.12)	0.44 (1617.85)	0.43 (1138.14)
	σ_{fm}^2	0.11 (221.85)	0.18 (300.76)	0.05 (186.02)	~ 0 (8.57×10^{-7})	0.03 (78.51)
	σ_e^2	0.16 (337.80)	0.11 (180.71)	0.12 (435.50)	0.24 (846.81)	0.21 (567.85)

Table S2: Summary of QTLs found for each model in each environment. "1" indicates that the QTL is associated to the flowering time in this environment with this model and "0" indicates no association.

	13EX01					13EX02					13EX03				
	A_{AIS}	$A_{XX'}$	AD	FM	FMI	A_{AIS}	$A_{XX'}$	AD	FM	FMI	A_{AIS}	$A_{XX'}$	AD	FM	FMI
FT09.199	0	0	1	0	1	0	0	0	0	0	0	1	1	0	1
FT11.47	0	0	0	0	0	0	0	1	1	1	0	0	0	0	0
FT16.167	0	0	0	0	0	1	1	0	0	0	0	1	0	0	0
FT01.98	0	0	0	0	0	0	0	0	0	0	0	0	0	0	0
FT15.102	1	1	0	1	0	1	1	0	0	0	1	1	0	0	0
FT02.78	1	1	0	0	0	1	1	0	0	0	0	0	0	0	0
FT17.184	0	0	0	0	0	0	0	0	0	0	0	0	0	0	0
FT05.208	0	0	0	0	0	0	0	0	0	0	0	0	0	0	0
FT04.144	0	0	0	0	0	0	0	0	0	0	0	0	0	0	0
FT07.34	0	0	0	0	0	0	0	0	0	0	0	0	0	0	0
FT17.13	0	1	0	0	0	0	0	0	0	0	0	0	0	0	0
FT04.74	0	1	0	0	0	0	0	0	0	0	0	0	0	0	0
FT13.190	0	1	0	0	0	0	0	0	0	0	0	0	0	0	0

	13EX04					13EX06				
	A_{AIS}	$A_{XX'}$	AD	FM	FMI	A_{AIS}	$A_{XX'}$	AD	FM	FMI
FT09.199	1	0	0	1	1	0	0	1	1	1
FT11.47	0	0	0	0	0	0	0	0	0	0
FT16.167	0	0	0	0	0	0	0	0	0	0
FT01.98	1	1	0	0	0	1	0	0	0	0
FT15.102	1	1	0	0	0	1	1	0	0	0
FT02.78	0	0	0	0	0	0	0	0	0	0
FT17.184	0	0	0	0	0	1	1	0	0	0
FT05.208	0	0	0	0	0	1	0	0	0	0
FT04.144	0	0	0	0	0	1	0	0	0	0
FT07.34	0	0	0	0	0	1	0	0	0	0
FT17.13	0	0	0	0	0	0	0	0	0	0
FT04.74	0	0	0	0	0	0	0	0	0	0
FT13.190	0	0	0	0	0	0	0	0	0	0
